# Supplementary figures and images for: Role of Maternal Antibodies in the Protection of Broiler Chicks against Campylobacter Colonization in the First Weeks of Life
Source: Animals (Basel). 2024 Apr 25;14(9):1291. doi: 10.3390/ani14091291 (PMC11083098; doi:10.3390/ani14091291)

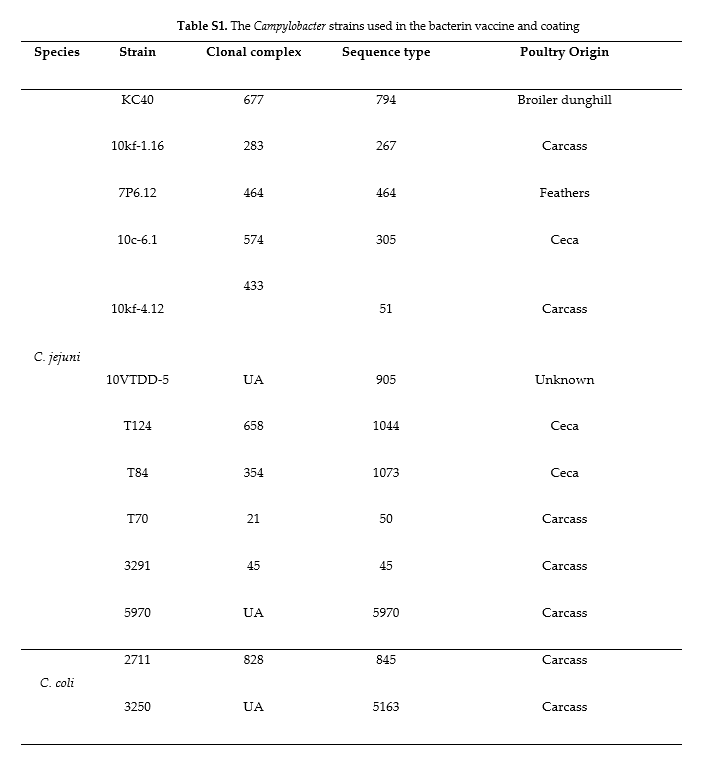

Supplement: Supplementary file 1 [file animals-14-01291-s001.zip › Table S1.PNG]

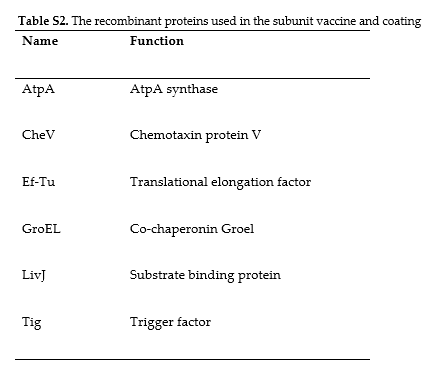

Supplement: Supplementary file 1 [file animals-14-01291-s001.zip › Table S2.PNG]

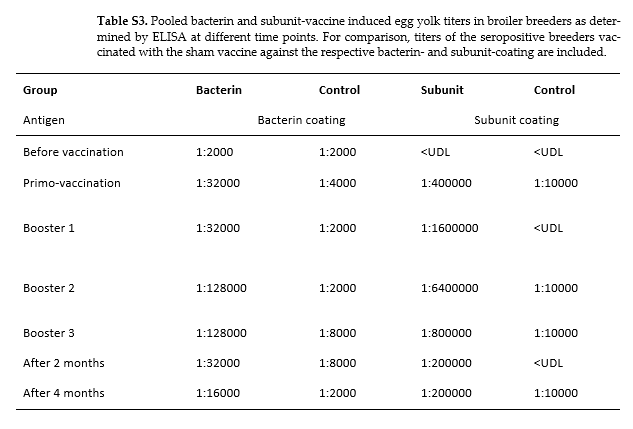

Supplement: Supplementary file 1 [file animals-14-01291-s001.zip › Table S3.PNG]
